# Insulin injection technique in the western region of Algeria, Tlemcen

**DOI:** 10.11604/pamj.2020.36.327.21278

**Published:** 2020-08-24

**Authors:** Mohammed Nassim Boukli Hacene, Meriem Saker, Amina Youcef, Soumia Koudri, Souad Cheriet, Hafida Merzouk, Ali Lounici, Nimer Alkhatib

**Affiliations:** 1 Department of Pharmacy, Faculty of Medicine, University Abou-Bekr Belkaïd, Tlemcen 13000, Algeria,; 2 Laboratory of Physiology, Pathophysiology and Biochemistry of Nutrition, Department of Biology, Faculty of Natural and Life Sciences, Earth and Universe, University Abou-Bekr Belkaïd, Tlemcen 13000, Algeria,; 3 Department of Internal Medicine, University Hospital Center Tidjani Damerdji Tlemcen 13000, Algeria,; 4 Laboratory of Research on Diabetes, Faculty of Medicine, University Abou-Bekr Belkaïd, Tlemcen 13000, Algeria,; 5 Center for Health Outcomes and PharmacoEconomic Research, College of Pharmacy, University of Arizona, Tucson, Arizona, USA,; 6 MidCapital Health Care, Amman, Jordan

**Keywords:** Diabetes, Insulin, insulin, injection technique, injection site, pen needle length

## Abstract

**Introduction:**

Algeria has more than 1.7 million diabetic patients on to whom a descriptive assessment particularly on the insulin usage behaviors has not yet been initiated, although is needed. This study aims to provide a descriptive analysis of how Algerian diabetic patients perceive and apply insulin injection techniques.

**Methods:**

using the “patient” questionnaire within the Injection Technique Questionnaire (ITQ) 2016 survey, this study assessed the insulin injection practices of 100 patients recruited over a seven-month period in western Algeria at the Tlemcen University Hospital Center. The results of this study are compared to those of the ITQ 2016 survey.

**Results:**

pens are the instruments of injection for 98% of Algerians who continue to use mostly long needles of 6- and 8-mm, although 4mm needles are the recommended safer option. Insulin analogues (fast and basal) are plebiscite. Arms and thighs are the preferred injection sites; the abdomen (the preferred site elsewhere) is neglected for reasons to be investigated. The correct re-suspension technique for cloudy insulin is unknown. Extensive pen needle re-use (10+ times) for over half of the patients exposes them to both higher intramuscular (IM) injection risk and lipohypertrophy (LH). Injection training is performed in Algeria by the diabetologist.

**Conclusion:**

this study describes for the first time Algerian patients´ insulin injection technique. It highlights their skills and identifies many deficiencies which patients and professionals must correct given the issues in this area.

## Introduction

The number of diabetic patients is constantly increasing globally. The prevalence of diabetes in Algeria grew by 6.9% in 2017. According to the International Diabetes Federation, this percentage translates to 1,782,000 diabetics [1]. Health professionals admit that diabetes pharmaco-therapeutic management is not optimal in Algeria and many gaps remain to be filled [2]. Amongst all other diabetes treatments, one that stands out as requiring special attention, due to it repelling a number of patients, is insulin. For patients with Type 1 diabetes, insulin is the standard treatment - an unavoidable drug. Insulins are also frequently used in Type 2 diabetes. Ideally, controlling insulin injection would simultaneously enable insulin to act effectively make possible achieving glycemic and therapeutic goals. Insufficient knowledge of all related key points of insulin injection technique leads to dissatisfaction, inconvenience and other complications. It is therefore important to identify the current practices of diabetics on insulin and to determine their knowledge of insulin injection technique. The fourth international study on insulin injection technique was conducted in 2014 to 2015 in 42 countries over at least a six-month period. Each country could have one or more centers and each center had to recruit at least 25 patients. The total number of participants exceeded 13,289 patients. Patients were asked about their insulin injection habits. Specific targeted questions covering all aspects of insulin injection, such as the choice of injection site and insulin needle length, were answered. In addition, psychological and social aspects, complications of insulin therapy and patients´ education were evaluated. Overall results were collected, analyzed and published [3,4]. They served as a source of information for the development of new insulin injection technique recommendations [5]. In conjunction, detailed results country-by-country were populated into an interactive format [6]. During the past three years, many countries have published their national data [7-13]. The number of countries participating in the international studies on insulin injection technique has steadily increased [3,14-16] although Algeria has never been one of them. Insulin injection technique of Algerian patients has never been evaluated by this initiative or by any other. This propelled the need to investigate Algerians´ experiences using insulins. This study aims to provide a cross-sectional investigation that describes Algerians´ insulin practices and recognizes deficiencies in the treatment of Algerian diabetic patients. Methods

## Methods

Algerian patients were recruited over seven months between 1^st^ October 2018 and 30^th^ April 2019. The study was performed at the Internal Medicine Department (inpatients) and the specialized polyclinic of Boudghene (day consultations), both of which are part of the Tlemcen University Hospital Center. The recruitment methodology previously described was scrupulously respected [3,9]. The inclusion criteria were restricted to patients with diabetes who have been on insulin for at least six months. The study was conducted in accordance with good clinical practice and the Helsinki Declaration. Ethical approval was not required. After explanation of the study objectives, patients gave verbal consent to be included. The fourth version of the ITQ was validated in 2014 and published in eight languages [3]. It consisted of two distinct parts: “Patient” and “Nurse” questionnaires. Only the 2016 Patient questionnaire was filled out in this study, the Nurse survey could not be explored for logistical and practical reasons. The questionnaire was presented to patients in both French and Arabic versions and whenever necessary, the interview was conducted in local dialectal Arabic. Algerian diabetics´ answers to our questionnaire were collected and analyzed on the Statistical Package of Social Science (SPSS) software version 17. As a result, our descriptive results and statistics are presented in this study and compared to the global results demonstrated by the international Injection Technique Questionnaire in 2016 (ITQ, 2016).

## Results

Of all the Algerian patients studied, 94% were adults and 38% were males. The average age, duration of diabetes and number of years on insulin were 46, 12.21 and 7 years respectively. Algerian patients´ characteristics are described, their identity and stratification according to the type of diabetes is presented in Table 1. The average daily dose of insulin for Algerian patients (Table 1) is slightly higher than that of the international population (+5.5 IU), as is the average daily dose of rapid insulin analogues (+13.5 IU). However, no nocturnal hypoglycemia (NPH) insulin patient was found in this study´s sample.

**Table 1:** population characteristics for Algerian and ITQ 2016 patients

Population characteristics		Algeria		Worldwide	
		Mean ± SD	N	Mean ± SD	N
Age		46.69 ± 18.13	100	51.9 ± 18.1	13225
BMI		26.68 ± 6.50	100	26.6 ± 6.2	12806
Years with DM		12.21 ± 7.79	100	13.2 ± 9.7	9197
Age DM diagnosed (y)		34.70 ± 16.99	100	39.9 ± 17.2	12737
Years taking pills		12.57 ± 7.35	44	8.3 ± 7.2	6607
Years taking insulin		7.00 ± 6.45	100	8.7 ± 8.9	8242
TDD of rapid insulin (IU)		31.00 ± 16.85	4	27.0 ± 20.7	1422
TDD of fast analogue (IU)		45.23 ± 21.17	61	31.9 ± 21.6	3467
TDD of NPH (IU)		/	0	31.6 ± 24.4	1134
TDD of basal analogue (IU)		23.62 ± 12.48	85	27.6 ±19.5	4709
TDD of premixes (IU)		43.71 ± 29.25	16	43.0 ± 25.3	1796
TDD of insulin (IU)		54.99 ± 32.94	100	48.5 ± 32.4	7756
HbA1c (%)		8.46 ± 1.96	100	8.47 ± 2.14	7663
HbA1c (%) for T1DM patients		9.11 ± 2.52	34	8.49 ± 1.9	2600
HbA1c (%) for T2DM patients		8.12 ± 1.51	66	8.48 ± 1.9	4840
HbA1c (%) for GDM patients		/	0	6.17 ± 1.3	80
Type of Diabetes	T1DM	34	34	34	2790
	T2DM	66	66	65	5378
	GDM	0	0	1	86
Patients´ Identity	Self-injecting adult (18 years old or older)	94	94	91	8627
	Self-injecting adolescent (13-17 years old)	4	4	5	469
	Self-injecting child (<13 years)	0	0	2	226
	Parent who gives injections to my child)	2	2	2	209

**Injection device:** only 2% of this survey´s population does not use pens as a means of insulin injection (Table 2).

**Insulin needle length and changing of length:** if 10% of Algerian patients have no idea of their insulin needle length, the preferred lengths in descending order are 6 mm (32%), 8 mm (25%), 5 mm (20%) and 4 mm (8%) (Table 2). Forty-two percent of Algerian patients state that they have not changed needle lengths since they started injecting insulin (Table 2).

**Table 2:** device, needle length characteristics and number of injections per day for Algerian and ITQ 2016 patients

Characteristics		Algeria		Worldwide	
		%	N	%	N
**Device**	Pen	98	98	86	11070
	Syringe	2	2	10	1238
	Pen and syringe	0	0	1	337
	Other (e.g. Pump)	0	0	3	184
**Pen needle length (mm)**	8	22.5	25	23	2280
	6	28.8	32	18	1825
	5	18	20	36	3659
	4	7.2	8	23	2345
**Pen needle length change**	Yes	42	42	/	/
	No	58	58	/	/
**Reason of Pen Needle Length Change**	To make injections more comfortable	37	18	/	/
	To reduce risk of going into muscle	8	4	/	/
	To reduce the risk of hypoglycemia	8	4	/	/
	Dont know	47	23	/	/
**Number of injections per day**	1	25	25	16	1523
	2	6	6	26	2480
	3	22	22	13	1240
	4	40	40	34	3213
	5	7	7	8	735
	6	0	0	2	197
	>6	0	0	2	141

**Number of daily injections:** an intensive therapeutic regimen consisting of at least 4 daily injections is undertaken by about 40% of Algerian population against 34% worldwide (Table 2).

**Injection sites:** about 85% of Algerians use thighs and arms to inject insulin, 64% use the abdomen and only 1% injects into the buttocks. The combination abdomen/thigh/arms is being adopted by almost half of Algerian participants (Table 3).

**Table 3:** injection sites used for Algerian and ITQ 2016 patients

Injection sites used		Algeria		Worldwide	
		%	N	%	N
**Preferred injection sites**	Abdomen	64	64	90.8	N/A
	Thigh	85	85	45.9	N/A
	Buttocks	1	1	14.1	N/A
	Arm	85	85	33.9	N/A
**Combinations of injection sites**	Abdomen alone	2	2	30.35	1995
	Thigh alone	6	6	4.07	268
	Arm alone	6	6	2.22	146
	Abdomen / Thigh	6	6	22.97	1510
	Abdomen / Arms	7	7	6.66	438
	Thigh / Arms	23	23	4.53	298
	Abdomen / Thigh / Arms	48	48	19.06	1253
	All 04 sites	01	01	10.10	664
	Total	100	100	100	6572

**Rotation:** about 75% of Algerian patients report rotations of their injection sites (compared to 83.9% worldwide). To the question: “How would you describe your rotations?”, 52% answered with the definition of a good rotation, as to inject insulin at least 1 cm away from the previous injection site, and 71% preferring alternating left-right (Table 3). It would seem that a large proportion of Algerian patients practice injection rotation correctly.

**Lipohypertrophy:** about 44% of the Algerian population complains of LH, localized (in decreasing order) in the arms (44%), thighs (34%), abdomen (20%) and the buttocks (2%). The vast majority of patients with LH deny injecting in these sites (80%) (Table 4).

**Table 4:** rotation and lipohypertrophy for Algerian and ITQ 2016 patients

Practices			Algeria		Worldwide	
			%	N	%	N
**Rotation Assessment**	**Rotation**	Yes	75	76	90	11711
		No	25	24	10	1270
	**Description**	I move back and forth from right side of my body to left	71	53	27	2267
		I inject about a finger's breadth (1cm) from where I previously injected	52	39	18	1508
		I move from an injection site to another	48	36	20	1705
		My injections describe a circle around my injection sites	4	3	9	722
		My injections describe lines across my injection sites	1	1	4	346
**LH**	**Presence**	Yes	44	44	29	3855
		No	56	56	71	9334
	**Localization**	Abdomen	20	10	60	2368
		Thigh	34	17	12	487
		Buttocks	2	1	1	46
		Arm	44	22	10	401
	**Injecting into LH**	Always	0	0	8	456
		Sometimes	20	8	60	3303
		Never	80	44	31	1708
	**Reason**	It is convenient	12.5	1	11	210
		It is less painful	0	0	17	313
		Just a habit (I always inject there)	37.5	3	31	574
		Do not know	50	4	28	523

**Insulin storage:** overall Algerian patients adhere to the correct practice of keeping unopened insulin in the refrigerator. Once opened, among the 45% of those who keep it cold after each injection, yet only 38% take the time to let it warm up before injecting. Algerians are falling short on this point compared to the international population (56.3%), (Table 5).

**Table 5:** some current practices for Algerian and ITQ 2016 patients

Current practices			Algeria		Worldwide	
			%	N	%	N
**Insulin storage**	**Before beginning to use the pen or vial**	Refrigerator	92	92	88.6	8566
		Room temperature	8	8	11.4	1104
	**After beginning to use the pen or vial**	Refrigerator	45	45	43	4131
		Room temperature	55	55	57	5479
	**Warm-up insulin kept in the refrigerator before injecting it**	Yes	38	17	56.3	4432
		No	62	28	43.7	3439
**Reconstitution of Cloudy Insulin**	**Before each use?**	Yes	87.5	14	65	5968
		No	12.5	2	24	2201
	**Number rolls/tips**	20 or more	0	0	12	385
		15	7.1	1	4	137
		10	35.7	5	35	1086
		6	14.3	2	4	134
		5	35.7	5	16	507
		2	7.1	1	7	230
**Dwell times after pen injection**		< 5 s	29	26	19	1612
		5-10 s	20	20	46	3954
		More than 10 s	25	25	32	2770
		'Not aware of how long'	26	26	4	337
**Pen Needle Re-use**	**Re-use**	Yes	98	98	56	6676
		No	2	2	44	5285
	**How many times**	2 times	2	2	31	1222
		3-5 times	17	17	40	1583
		6-10 times	29	28	16	639
		More than 10 times	52	51	14	541

**Re-suspending cloudy insulin:** no patients in this study´s sample adequately follows the recommendations about this crucial step of reconstitution for nocturnal hypoglycemia (NPH) and premix insulin (Table 5).

**Hold time of the needle under the skin:** when asked how long they held the insulin needle under the skin, only a quarter of Algerian respondents respected the recommendation, leaving it at least 10 seconds before withdrawing it, compared with 32% of the worldwide population and 48.3% of Turkish patients. In addition, 26% of this study´s population did not know how long to leave the needle under the skin. This could potentially highlight a lack of knowledge about the length of time it takes for the full dose of insulin to be administrated (Table 5).

**Re-using pen needles:** with the exception of two, all Algerian patients reuse their pen needles: half more than 10 times and about 26% between 6-10 times (Table 5). The reason given for this reuse is pecuniary (68%) but also due to the non-availability of needles (61%).

**Injection site inspection:** another issue of note surrounding Algerian diabetes patients´ injection habits is the follow-up of patients under insulin (Table 6): 82% do not remember the last time they had their injection site inspected, compared with 39% worldwide. Most patients who have their administration site inspected report that this is happening regularly; every three months.

**Table 6:** injection inspection and training for Algerian and ITQ 2016 patients

			Algeria		Worldwide	
			%	N	%	N
**Injection site inspection**	**Frequency**	Routinely every visit	13	13	28	3537
		Once a year	2	2	13	1581
		Only if I complain of a problem at a site	3	3	20	2527
		I cannot remember my sites ever being checked	82	82	39	4860
	**Every visit, that it is to say every**.	1 month	0	0	11	297
		2 months	0	0	8	214
		3 months	92.3	12	50	1347
		6 months	7.7	1	20	545
**Injection instructor**		General Nurse	10	10	23	2161
		Diabetes Nurse	4	4	47	4411
		Diabetes Educator	3	3	12	1159
		Doctor (General Practitioner)	8	8	5	478
		Doctor (Diabetes Specialist)	71	71	10	941
		Pharmacist	4	4	2	191
		A representative of the pen or needle manufacturer	0	0	1	99

**Source and training needs:** according to the ITQ 2016 results and those of countries which have published their statistics, a nurse is most often (50%-70%) the health practitioner who explains insulin injection technique to the patient. In Algeria, a nurse is involved in this education for only 14% of patients, as the majority is educated by the diabetes specialist (71%) (Table 6).

## Discussion

After Strasbourg in 1997 [17], Barcelona in 2000 [18] and Athens in 2009 [19], experts from all over the world gathered in Rome in 2015 to update recommendations for insulin injection technique at the FITTER summit [5]. These recommendations were subsequently enriched and many countries developed their own recommendations [20-22]. It is on the basis of the Rome summit´s conclusions that the results of this study will be discussed here. Almost all Algerian patients inject insulin using pens (98%), in line with the global international movement to transition from using syringes to using pens. Slight differences with international results may be explained by the fact that syringes continue to be the preference in some countries, such as Brazil [7] and India [9], and by the relatively recent introduction of insulin pumps in Algeria. The use of these devices is still limited to specific departments of the largest Algerian hospitals and are not reimbursed, as well as their consumables [23]. Patients´ habits around the world have been challenged by the 2010 introduction of the 4 mm needle associated with lower risks of IM injection and hypoglycemia, as well as by most recent recommendations for its advocacy for reducing the risk of muscle injury [5]. Indeed, as recent as 2010, nearly half of the world´s population preferred the 8 mm needle, but today shorter needle lengths are as popular [3,16]. The findings of this study suggest that the 4 mm needle has difficulties in gaining market share in Algeria (8%), with Algerians preferring 6 mm (32%), 8 mm (25%) and 5 mm (20%) needles.

Smith *et al*. explained that a therapeutic patient´s education program involving the switch to 4 mm needles would significantly reduce their total daily dose of insulin by approximately 6 IU/pt/day; a direct saving of £42 million annually for the English and Irish National Health Service. Additionally, indirect savings are obtained by improved HbA1c results and reduction of the pathogenesis of diabetes complications and of hospitalizations [24]. The fact that more than half (58%) of Algerian patients are not changing the pen needle length they use since beginning to use insulin is a marker that must be questioned. It emphasizes that initial choice of needle length is crucial since the patient is likely to continue using it for a long period of time. It underlines the importance of the health professional´s educative role during insulin initiation. With the abdominal region being wide, it allows for easy rotations and is associated with reduced risk of LH [25]. It is also the injection site where insulin is the most rapidly absorbed and is therefore the recommended site for the administration of human soluble insulin. Several reasons may explain that arms are not the preferred site for insulin injection: reduced surface area, difficulty in reaching these regions oneself, higher risk of IM injection, lower thickness of the subcutaneous tissue and greater difficulty for rotations. Also, when injecting insulin into arms, it´s recommended to use needles of 6 mm and longer to practice skinfold which implies that the injection must be administered by a third party [5]. The abdomen was and continues to be the most commonly used site for insulin injection [3]. But, intriguingly, it occupies neither the first nor the second position of Algerians´ preferred injection sites and is preceded by arms and thighs. Algerian patients complain more about LH than patients worldwide (44% versus 29%). The abdominal region is the preferred region for insulin injection around the world, with LH being more common there than elsewhere [4]. Algerian patients prefer arms, and declare having the most LH in these areas. Of particular note in this study is Algerians´ preferential insulin injection site. Future investigations should examine reasons that led to this rejection of the abdomen in favor of arms, increasing the risk of LH and IM injection. In Algeria, it is primarily the diabetologist who educates the patient about insulin injection technique. In the future, this specialist will not have sufficient time to carry out this practice given the ever-increasing number of patients on insulin. Algerian nurses will have to occupy this ground, as is the case worldwide [4] and will be responsible for advising and reviewing injection technique. Algerian health organizations should facilitate to (re)upgrade their knowledge in this area [26].

Pen needle reuse is not an exclusively Algerian, but a global practice [3]. However, as patients worldwide reuse needles mostly 3-5 times (39.7%), Algerian patients appear to be worse on this point with 52% tending to over-reuse (10+ times). The financial aspect cannot alone justify this bad habit. Ignorance of the dangers of this practice (under-dosage, overdoses, LH, pain, etc.) is a reality for many. The psychological dimension of the injection technique was assessed in an editorial in 2016. According to the assessment, it is easier to convert a patient to a 4 mm needle or to abandon injection through clothes, but the problem of pen reuse appears to be more problematic. Patients may be in the habit of doing this and/or be unable to finance disposable needles and even reluctant to stop reusing needles. It is recommended to schedule some time to discuss the advantages and disadvantages of the patient´s practices, to find pragmatic solutions in line with his knowledge and attitudes, and to include with the patient in the final decision [27].

The reimbursement system of pen needles differs from country to country, these being entirely the responsibility of patients in Algeria. A Chinese study analyzed habits and attitudes of patients covered by insurance and those who were not. Differences were noteworthy. Those who were not covered by the reimbursement system and who therefore had to purchase the needles from their own pocket had more LH and reused their needles more. In addition, their insulin cost them significantly more in 6 months as their total daily dose (TDD) of insulin was higher and they were 4.6 times more likely to have higher care costs [28]. Algerian health insurance companies would be gaining two-fold if there was a change in reimbursement policy, by easing the economic burden of pen needles for its taxpayers. Additionally, it would encourage patients to adopt better health practices and to increase their satisfaction. This would retroactively achieve quantifiable indirect cost savings, certainly greater than the initial savings. Re-suspension of insulin is not necessary for clear Aspart or Glargine insulins. Neglecting this step would expose patients, with NPH-type or mixed insulins, to large dose errors and thus to risks of hypo- or hyperglycemia. Recommendations advocate slowly shaking and/or inverting cloudy insulin more than 20 times in total prior to use [5]. No patient in this study adopts the correct practices regarding reconstitution of these insulins, whereas around 10% around the world do.

Therapeutic education directed specifically at this point of re-suspending cloudy insulins should be undertaken to decrease the worrisome number of users not respecting the recommendations. An awareness-raising approach for the need to systematically reconstitute NPH or mixed insulin and learning good practices to adopt: shake slowly and/or roll in the hands cloudy insulin ideally more than 20 times will help avoid dosing errors. Algerian health professionals should pay greater attention to the inspection of insulin injection sites. This would certainly help to detect earlier and more effectively, and to correct some attitudes and complications such as LHs, LH injections, inadequate rotations, insulin leaks and bleeding. The result would be a lower frequency of these conditions for patients and an improvement in their quality of life.

**Limitations of the study:** some limitations to this study must be recognized: firstly, its monocentric character. The study was conducted at only one hospital: the Tlemcen University Hospital Center. While it is a hospital that funnels a large number of patients from all over the western region of Algeria, the conclusions made from this study cannot be generalized to the entire Algerian population on insulin. Secondly, the size of the sample - 100 patients - although greater than that of many centers that participated in the international study, is insufficient to claim that the conclusions of the study are representative of the whole Algerian population who use insulin. Thirdly, the nurse questionnaire of the ITQ 2016 was not covered by our study. This did not allow us to verify and corroborate certain information given by patients with nurse observations, such as the presence and injection into LHs and skinfold practice. Additionally, it was not possible to know whether the patient was subject to frequent unexplained hypoglycemic episodes or hypoglycemic variability. Further studies are needed to address these points. This study evaluated for the first time Algerians´ insulin injection technique according to the model and methodology of the ITQ 2016. The question “how does the Algerian patient inject with insulin?” is no longer unanswered. This study provides elements of information likely to facilitate better management of the Algerian diabetic patient.

## Conclusion

This study details the habits and attitudes of Algerian patients concerning the technique of insulin injection. Habits which, until now, have never been investigated in detail. Many of the practices of Algerian insulin injectors have been revealed. The Algerian patient uses the pen, continues preferring the old long pen needles, injects himself into the arms and thighs more so than into the abdomen, over-reuses needles that he puts directly into the general waste, and continues to be trained on injection technique by diabetologists. This study highlights misunderstandings and the lack of competence of Algerian diabetics regarding insulin injection technique. The alarming figures found should encourage health professionals to: propose a basic therapeutic education directed to details of the injection technique; systematize this approach for all diabetics under insulin; re-propose, afterwards, on a regular basis, a thorough therapeutic education to consolidate gains and fill in persistent gaps.

### What is known about this topic

- Insulin injection technique has been evaluated previously around the world, particularly in industrialized countries, never in Algeria;
- Mastering every detail of the technique is a prerequisite for the efficacy and safety of insulin treatment.

### What this study adds

- This study is the first to evaluate insulin injection technique among Algerian patients, highlighting their skills and identifying many deficiencies in this domain;
- The results obtained will improve the management of an endemic disease in Algeria, diabetes.
